# Pancreatoduodenectomy for Periampullary Tumors Presenting with Acute Pancreatitis

**DOI:** 10.1155/2020/7246895

**Published:** 2020-02-28

**Authors:** Xuefeng Cao, Xixiu Wang, Xiaoliang Xu, Yanmin Lu, Baolei Zhao, Xingyuan Zhang, Qiangpu Chen

**Affiliations:** ^1^Department of Hepatobiliary Surgery, Binzhou Medical University Hospital, Binzhou, Shandong Province, China; ^2^Department of Cardiovascular Medicine, Binzhou Medical University Hospital, Binzhou, Shandong Province, China; ^3^Pediatric Surgery, Binzhou Medical University Hospital, Binzhou, Shandong Province, China; ^4^Nutriology Department, Binzhou Medical University Hospital, Binzhou, Shandong Province, China

## Abstract

**Background:**

Periampullary tumors (PT) may rarely present as acute pancreatitis (AP) or acute recurrent pancreatitis (ARP). Unlike other cases of AP and ARP, these conditions necessitate pancreaticoduodenectomy (PD), and timely diagnosis is crucial. *Materials and Methods*. A retrospective review of clinical, radiological, surgical, and pathological data was conducted for patients admitted to the Binzhou Medical University Hospital during the period from January 2010 to December 2017, for AP or ARP caused by PT. All patients included in the study group had undergone PD. The perioperative data for these patients was compared with data for patients with PT but without AP or ARP who underwent PD during the same period (control group).

**Results:**

During the study period, 412 patients with AP or ARP were treated; among this group, 15 patients had PT. Compared with controls, patients in the study group were younger in age and had a longer course of disease, more frequent hospitalizations, and more severe derangements in laboratory data (*P* < 0.05). Operative time and intraoperative blood loss were significantly higher in the study group, but the incidence of postoperative outcomes such as pancreatic/biliary fistula, abdominal infection, postoperative hospital stay, and mortality were similar between groups (*P* < 0.05). Operative time and intraoperative blood loss were significantly higher in the study group, but the incidence of postoperative outcomes such as pancreatic/biliary fistula, abdominal infection, postoperative hospital stay, and mortality were similar between groups (

**Conclusions:**

Neither AP nor ARP has any adverse impact on the outcomes of PD. However, in the treatment of younger patients suffering from AP or ARP, unexplained pancreatic duct dilation and weight loss should raise the suspicion of PT. EUS and EUS-FNA may be helpful in making the diagnosis.

## 1. Introduction

Acute pancreatitis (AP), an inflammatory disorder of the pancreas, is one of the leading causes of hospital admission for gastrointestinal disorders in the USA and many other countries [1]. Acute recurrent pancreatitis (ARP), defined as the occurrence of two or more episodes of AP, generally occurs in the setting of a normal morphofunctional gland [2]. In the USA and other western countries, the main causes of AP and ARP are gallstones and alcohol abuse. In China, the main cause is choledocholithiasis. Other likely risk factors include genetics, drugs, and smoking [3]. However, a small subset of these patients may have periampullary tumors (PT) causing pancreatic duct obstruction leading to AP or ARP. Although the symptoms of AP or ARP are mild in patients with PT, early detection of PT is crucial, as radical surgery is the only curative treatment. Delayed diagnosis can lead to future attacks of AP and disease progression, both of which contribute to morbidity and mortality. In this study, we reviewed cases of PT with an initial presentation of AP or ARP in patients who subsequently underwent pancreatoduodenectomy (PD). The aim of the study was to analyze the clinical, biochemical, and radiological characteristics of these cases and associated postoperative outcomes.

## 2. Material and Methods

This retrospective, single-center study was conducted during the period from January 2010 to December 2017 at the Binzhou Medical University Hospital, Shandong province, China. The study was approved by the institutional ethics committee of the Binzhou Medical University Hospital. All procedures were performed in accordance with hospital regulations. Written informed consent was obtained from each patient.

A retrospective review of the electronic database was performed to identify patients who were eligible for inclusion in the study using the following inclusion criteria:

- Patients with AP or ARP due to PT who had undergone computed tomography (CT) or magnetic resonance imaging (MRI)
- Patients who underwent PD for PT
- Patients with serum amylase levels elevated >3 times normal

Exclusion criteria were as follows:

- Patients who did not undergo surgery for PT
- Patients with AP or ARP who underwent surgery for a non-periampullary tumor
- Patients with PT and AP or ARP who underwent surgery other than PD
- Patients who received neoadjuvant treatment preoperatively, as it could introduce bias in the results
- Patients who underwent other abdominal procedures or operations before PD such as endoscopic ultrasound (EUS), endoscopic retrograde cholangiopancreatography (ERCP), and cholecystectomy

**Figure 1:**
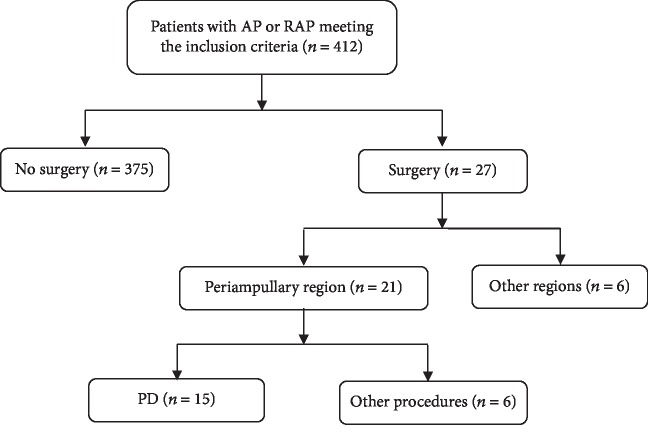
Flow chart depicting study design.

The flow chart for selection of the study population is presented in Figure 1.

All relevant clinical, inpatient laboratory, and radiological data were collected, including age, sex, course of disease, predisposing factors, clinical manifestations, radiological findings, inflammatory markers (e.g., white blood cells (WBC)), markers of liver function (e.g., total bilirubin (TBIL) and direct bilirubin (DBIL)), prealbumin (PA), albumin (ALB), nutritional status, tumor markers (e.g., carbohydrate antigen 19-9 (CA19-9)), calcium serum (Ca), and serum amylase (AMY). Perioperative and pathological data were also collected.

For comparison, patients who underwent PD due to periampullary tumors during the study period, without the clinical presentation of AP or ARP, were identified from the electronic medical records (control group). Demographic characteristics and surgical data for these patients are presented in Table 1.

Before the operation, patients in the study group were initially treated with nutritional support and medical therapy for AP until symptoms were relieved and AMY decreased to normal. All PD procedures in both groups were performed by a single surgical team. An upper midline abdominal incision was made, and the standard Whipple PD was performed. Pancreatojejunostomy was performed using the technique of duct-to-mucosa anastomosis. Postoperative care included antibiotics, analgesics, early ambulation, and nutritional support.

### 2.1. Statistical Analysis

Data were analyzed using Statistical Package for Social Sciences (SPSS) software, version 24.0 (IBM Co, Armonk, NY, USA). Data are expressed as mean ± standard deviation (SD), and incidences are reported as percentages. Categorical variables were compared using a chi-square test or Fisher's exact test. Continuous data were analyzed using Student's *t*-test (for independent samples) or the Mann–Whitney *U* test, depending on the normality of the data distribution. *P* value < 0.05 was considered to be statistically significant.

## 3. Results

During the study period, 412 patients were admitted to our institution for AP or ARP. Twenty-seven (6.55%) patients received surgical treatment: 21 (5.10%) patients with AP or ARP had PT without gallstones, alcohol intake, or hypertriglyceridemia, including 6 patients (1.46%) who underwent palliative operations because of an advanced tumor stage and 15 patients (3.64%) who underwent PD. These 15 patients were included in the study group (Table 2). There were 10 males (66.67%) and 5 females (33.33%), with a mean age of 49 years (SD, 10.39 years; range, 32–69 years). During the study period, 142 patients underwent PD for PT without AP or ARP (control group). The mean age of patients in the control group was significantly greater than the mean age of patients in the study group (58.46 (range, 28 to 79) years) (*P* < 0.05).

**Figure 2:**
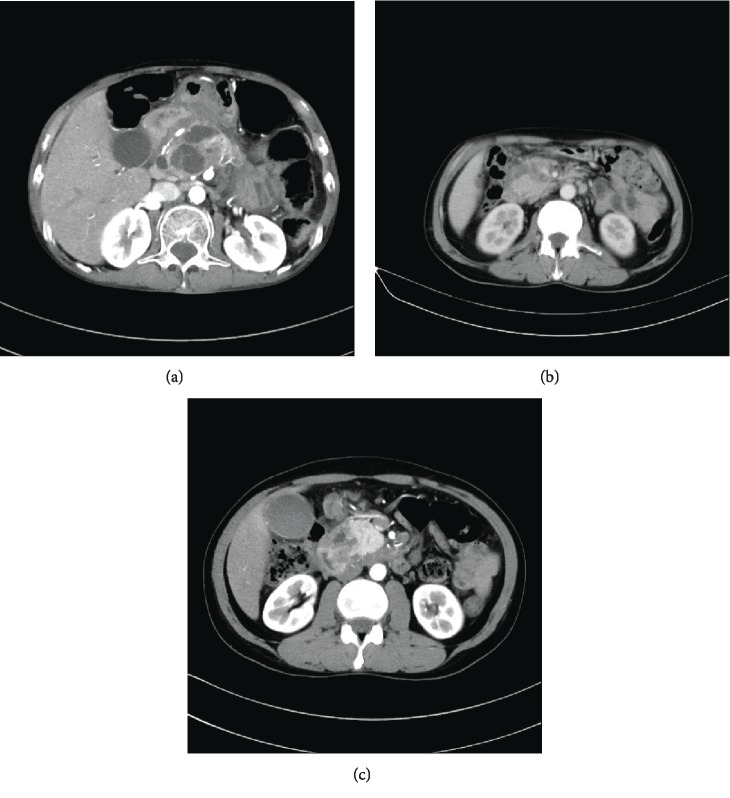
(a) IPMN of periampullary region. (b) Pancreatic adenocarcinoma of periampullary region. (c) Carcinoma of duodenum.

**Figure 3:**
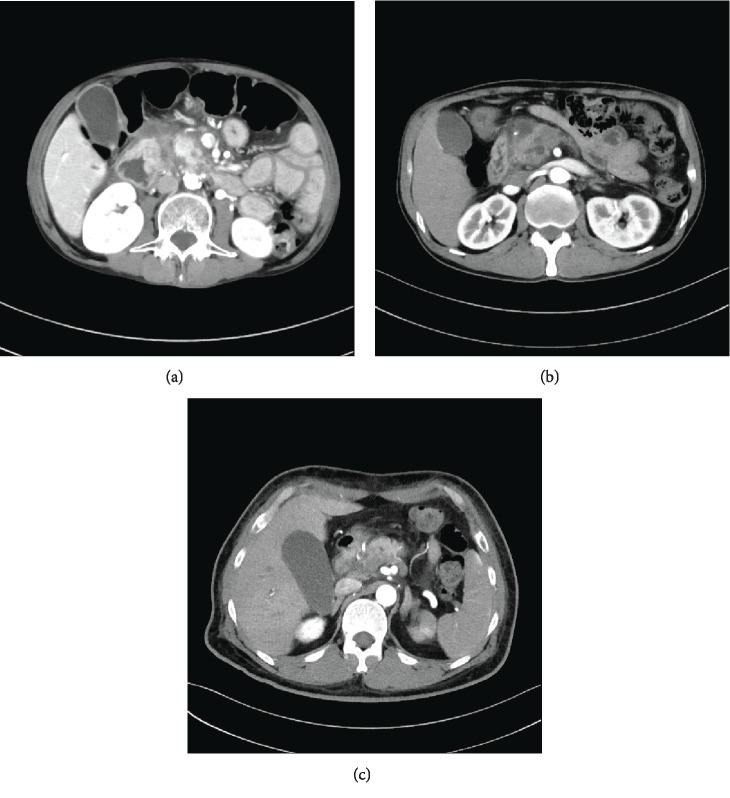
(a) Adenoma of duodenum. (b) Pancreatic cystadenocarcinoma. (c) Chronic pancreatitis with mass in pancreas head.

In the study group, the most common pathology was intraductal papillary mucinous neoplasm (IPMN), including 3 patients with main-duct type disease, 2 patients with branch-duct type disease, and 2 patients with mixed-type disease. The second most common pathology was pancreatic ductal adenocarcinoma. In the control group, the most common pathology was cholangiocarcinoma, followed by pancreatic ductal adenocarcinoma (Figures 2(a) and 2(b)). Other pathologies in patients with PT included pancreatic cystadenocarcinoma (Figure 3(b)), duodenal adenocarcinoma (Figure 2(c)), duodenal adenoma (Figure 3(a)), and chronic pancreatitis with head mass (Figure 3(c)). In the study group, all the patients had resection margins free of tumor.

For patients in the study group, the most common presentation was abdominal pain (86.67%). In the control group, the most common presentation was painless progressive obstructive jaundice (76.06%) (Table 2). Among patients in the study group, abdominal pain was dull in nature and mild in intensity. As the pain is often attributed to AP or ARP, the PT was often overlooked, and the diagnosis is delayed. Compared to patients in the control group, patients in the study group had a longer course of disease and a greater number of hospitalizations before final diagnosis (Table 2). In about two-thirds of cases (10/15), the second most common symptom was weight loss.

Laboratory blood analysis revealed that levels of TBIL, DBIL, PA, ALB, Ca, and CA19-9 were significantly higher in the control group, while levels of inflammatory markers such as WBC and AMY were significantly higher in the study group (*P* < 0.05) (Table 2). EUS/EUS-FNA was performed in 9 out of 15 patients (60%). The diagnostic accuracy of EUS-FNA was 77.78% (7/9).

Mean operative time (8.81 ± 1.37 h vs. 6.27 ± 1.90 h, *P* < 0.001) and estimated intraoperative blood loss (483.33 ± 123.44 mL to 312.32 ± 222.75 mL, *P* = 0.004) were significantly higher in the study group, compared with the control group. However, no significant difference was observed between groups in the rate of pancreatic/biliary fistula, rate of abdominal infection, postoperative hospital stay, or mortality (Table 2).

At 3 months follow-up after surgery, clinical examination, AMY, and abdominal CT showed that no patient had suffered a new attack of AP or ARP.

## 4. Discussion

In clinical practice, the most common symptom of periampullary tumor is painless, progressive obstructive jaundice [4, 5]. Uncommonly, PT can lead to AP or ARP and present with abdominal pain rather than jaundice [6–15]. In this study, we found that 21/412 (5.1%) patients with AP or ARP had PT. Because of this unusual presentation, the diagnosis of PT was delayed for up to 395 days, and 6 of 21 patients (28.57%) developed locally advanced periampullary carcinoma that necessitated palliative surgery, rather than PD. Hence, detailed investigations should be performed to rule out PT in patients with AP or ARP.

The prevalence of pancreatic cancer in individuals presenting with AP ranges from 2.6 to 13.8% [6–8]. The reported prevalence of AP in IPMN ranges from 12% to 67% [9–11]. Tumors of the duodenum, including adenoma and adenocarcinoma, rarely cause AP or ARP and have mainly been published as case reports [12–15]. In this study, we had similar observations, with IPMN being the most common cause of AP. The most common symptoms included abdominal pain, nausea and vomiting, and weight loss accompanied by localized inflammation of the periampullary region with dilation of the main pancreatic duct on radiology.

The exact mechanisms by which PT causes AP or ARP are not clear. It is believed to be caused by obstruction of the main pancreatic duct following tumor invasion or secretion of thick mucus by tumor cells. Obstruction of the man pancreatic duct leads to ductal hypertension and premature release and activation of pancreatic enzymes [16–18]. Additionally, ischemia secondary to vascular occlusion (caused by malignant cells or activation of pancreatic enzymes by tumor cells) may also lead to a blockage of the main pancreatic duct [18–20].

Endoscopic ultrasonography (EUS) combines the advantages of endoscopy and ultrasound and makes it possible to visualize the entire pancreas, neighboring blood vessels, and the common bile duct from various scanning positions in the stomach and duodenum [21]. Thus, it is one of the most accurate methods for the diagnosis and staging of pancreatic disease and is useful for the evaluation of PT [22]. Additionally, endoscopic ultrasonography–fine-needle aspiration (EUS-FNA) helps in obtaining cytological samples from pancreatic lesions, thus making pathologic diagnosis possible [23]. Previous studies have reported high sensitivity (slightly over 85%) and specificity (up to 100%) for the diagnosis of PT, especially pancreatic adenocarcinoma [24–27]. In this study, we found that about three-fourths of patients were accurately diagnosed by EUS/EUS-FNA. Thus, EUS/EUS-FNA is recommended for investigating whether AP or ARP is caused by PT.

CA19-9, a derivative of sialic acid, has been used widely as a diagnostic and prognostic marker for pancreatic cancer since the early 1980s [28]. Its sensitivity and specificity for the diagnosis of pancreatic cancer range from 80% to 85% and from 85% to 90%, respectively [29]. However, in patients with pancreatitis, serum CA19-9 may be elevated, even in the absence of malignancy. So, caution needs to be exercised in the interpretation of CA19-9 as a marker for differentiating carcinoma from pancreatitis. In a study by Bedi et al., which was aimed at assessing the value of CA19-9 in patients with chronic pancreatitis (CP) and pancreatic tumor, levels in excess of 300 U/mL suggested malignancy with 100% specificity [30]. In the present study, the value of CA19-9 was significantly higher in the control group. However, it was also high (>37 U/mL) in the study group in one-third of cases (5/15) and was helpful to investigate the diagnosis of PT or cancer.

PD is associated with high surgical morbidity (38-58%), prolonged hospital stay, and mortality [31, 32]. In our study, patients who presented with AP due to PT had significantly higher operative time and blood loss, compared to patients in the control group (Table 2) (*P* < 0.05). This was because of the presence of peripancreatic inflammation and adhesions due to prior AP, which rendered surgery more difficult technically. However, postoperative outcomes (including postoperative complications) were similar between groups (*P* < 0.05). A previous study by Chen et al. found that AP may significantly increase the incidence of severe complications and lengthen hospital stay following PD [33]. Possible reasons for the improved outcomes observed in our study include pancreatic duct size > 5 mm, the hard consistency of the pancreatic parenchyma due to ductal obstruction, and AP, which reduced the risk of pancreatic fistula [34].

This study has some limitations. First, this study was a retrospective single-center study. Second, the sample size was small. Future prospective studies with larger sample size are required to validate the findings of this study. Third, we included pancreatic carcinoma, IPMN, duodenal adenoma, and duodenal adenocarcinoma in the present study, which rendered the study group heterogeneous. However, due to the low incidence of PT presenting as AP, it was difficult to analyze the pathology of PT in isolation.

In summary, this report describes the clinical characteristics and surgical outcomes of patients with PT with an initial presentation of AP or ARP. Although AP and ARP remain rare presentations of this tumor, detailed investigations must be performed to avoid delayed diagnosis. Patients with PT with AP or ARP were younger in age, had unexplained dilation of the pancreatic duct, and had weight loss, compared with the patients in the control group. EUS/EUS-FNA may be helpful in detecting these tumors in the presence of AP. PD remains the preferred treatment for these patients with PT. Although the duration of surgery and estimated intraoperative blood loss were higher in the study group, the incidence of postoperative outcomes including the rate of pancreatic/biliary fistula, abdominal infection, postoperative hospital stay, and mortality was similar to those observed in patients without AP or ARP.

## Figures and Tables

**Table 1:** Demographic characteristics for patients in the study group.

No.	a	b	c	d	e	f	g	h	i	j	k	l	m	n	o	p	q	r
1	M	44	395	8	①	①②③④	5.73	8.2	2.9	0.16	38.8	2.39	339.9	6.82	Y	Incline to intraductal papilloma of the pancreas	①	—
2	M	33	180	3	①	②③④	4.78	13.4	3.7	0.3	36.3	2.25	395.3	6.15	N	—	①	—
3	M	69	120	2	①	①③	7.88	7.8	2.3	0.15	29.8	2.17	437.1	17.82	Y	Chronic inflammation with fibrous tissue hyperplasia	①	—
4	F	58	60	1	②	①	8.46	10.6	3.5	0.11	24.1	2.08	440.2	56.26	Y	Adenocarcinoma	②	T_3_N_1_M_0_
5	M	32	150	3	①	①③	9.95	9.9	2.9	0.09	26.8	1.68	378.5	51.76	N	—	⑤	T_3_N_0_M_0_
6	F	43	210	3	②	①②③	6.84	26.2	7.8	0.08	26.7	2.01	400.2	33.83	N	—	①	—
7	F	50	60	1	③	①⑤	11.2	7.1	2.9	0.19	33.8	1.88	3127.7	17.20	N	—	①	—
8	M	51	120	2	①	①③	9.22	8.6	3.2	0.17	34.5	2.26	470.8	22.71	Y	Duodenal papillary adenoma	④	—
9	F	48	30	3	⑤	①③⑥	12.1	17.6	4.5	0.21	36.2	1.74	330.3	27.14	Y	Duodenal adenoma with high grade intraepithelial neoplasia	③	T_3_N_1_M_0_
10	M	49	20	4	④	①②③	10.05	29.2	7.6	0.16	34.4	2.18	379.9	129.33	Y	Adenocarcinoma	②	T_3_N_0_M_0_
11	M	65	60	1	②	①⑤	11.67	33.7	10.5	0.09	25.4	1.96	550.7	163.12	Y	Adenocarcinoma	②	T_3_N_1_M_0_
12	M	46	90	2	①	①	7.8	10.3	4.2	0.14	30.2	1.96	688.8	11.46	N	—	①	—
13	M	43	90	2	⑤	①③	12.85	29.1	13	0.12	36.1	2.26	2058.6	59.61	Y	Blood clot and a few broken glandular epithelia	②	T_2_N_1_M_0_
14	F	59	60	3	③	①	9.55	17.6	10.4	0.11	33.2	2.02	468.1	17.62	N	—	①	—
15	M	45	120	4	⑤	③⑥	11.57	36.2	20.6	0.16	37.7	1.98	422.6	35.24	Y	Adenocarcinoma of duodenum	③	T_3_N_0_M_0_

Notes a: gender (male/female); b: age (years); c: course of disease (days); d: frequency of hospitalization; e: predisposing factors (① alcohol misuse, ② gallstones, ③ hyperlipidemia, ④ overeating, and ⑤ none); f: clinical manifestation (① abdominal pain, ② obstruction of upper alimentary tract, ③ weight loss, ④ dehydration, ⑤ abdominal bloating, ⑥ nausea and vomiting, ⑦ radiating pain (back or other), and ⑧ other); g: white blood cells (WBC, ×109); h: total bilirubin (TBIL, *μ*mol/L); i: direct bilirubin (DBIL, *μ*mol/L); j: prealbumin (PA, g/L); k: albumin (ALB, g/L); l: calcium serum (Ca, mmol/L); m: serum amylase (AMY, IU/L); n: carbohydrate antigen (CA19-9, U/mL); o: endoscopic ultrasonography (EUS, YES/NO); p: endoscopic ultrasonography–fine-needle aspiration (EUS-FNA)/endoscopic biopsy; q: postoperative pathology (① IPMN, ② pancreatic ductal adenocarcinoma, ③ adenocarcinoma of duodenum, ④ adenoma of duodenum, and ⑤ pancreatic cystadenocarcinoma); r: pathological stage (TNM).

**Table 2:** Comparison of demographic characteristics and surgical data between groups.

	Study group (*n* = 15)	Control group (*n* = 142)	*P* value
Age (years)	49 ± 10.39	58.46 ± 9.94	0.001
Gender			
Male (%)	10 (66.67%)	79 (55.63%)	
Female (%)	5 (33.33%)	63 (44.37%)	
Course (d)	117.67 ± 93.52	15.98 ± 15.65	0.001
Hospitalization frequency	2.80 ± 1.74	1.26 ± 0.46	0.004
Main clinical manifestation (%)	Abdominal pain (86.67%)	Obstructive jaundice (76.06%)	
Laboratory examination			
White blood cell (×109)	9.31 ± 2.39	7.01 ± 2.30	<0.001
Total bilirubin (*μ*mol/L)	17.7 ± 10.35	120.04 ± 96.32	<0.001
Direct bilirubin (*μ*mol/L)	6.67 ± 5.13	60.91 ± 56.89	<0.001
Prealbumin (g/L)	0.15 ± 0.06	0.21 ± 0.04	<0.001
Albumin (g/L)	32.27 ± 4.75	36.94 ± 2.20	0.002
Serum calcium (mmol/L)	2.05 ± 0.20	2.28 ± 0.13	0.001
Serum amylase (IU/L)	725.91 ± 789.51	43.01 ± 18.39	0.005
CA19-9 (U/L)	43.74 ± 45.37	376.52 ± 416.56	<0.001
Surgical data			
Duration of surgery (h)	8.81 ± 1.37	6.27 ± 1.90	<0.001
Blood loss (mL)	483.33 ± 123.44	312.32 ± 222.75	0.004
Pancreatic fistula (grade A/B)	2 (13.33%)	24 (16.90%)	0.724
Biliary fistula	1 (6.67%)	8 (5.63%)	0.873
Abdominal infection	3 (20.00%)	24 (16.90%)	0.762
Postoperative length of stay (d)	16.67 ± 5.01	20.04 ± 9.96	0.199
Mortality in 30 d (%)	0	7 (4.93%)	0.379
Postoperative pathology			
Pancreatic ductal adenocarcinoma	4 (26.67%)	35 (24.65%)	
Cholangiocarcinoma	0	38 (26.76%)	
Ampullary adenocarcinoma	0	29 (20.42%)	
Carcinoma of duodenum	2 (13.33%)	14 (9.86%)	
Adenoma of duodenum	1 (6.67%)	8 (5.63%)	
IPMN	7 (46.67%)	10 (7.04%)	
Pancreatic cystadenocarcinoma	1 (6.67%)	3 (2.11%)	
Others	0	5 (3.52%)	

## Data Availability

The data-sets generated and analyzed during the present study are available from the corresponding author on reasonable request.
